# Imaging inside highly scattering media using hybrid deep learning and analytical algorithm

**DOI:** 10.1002/jbio.202300127

**Published:** 2023-07-19

**Authors:** Ben Wiesel, Shlomi Arnon

**Affiliations:** ^1^ Ben‐Gurion University of the Negev, Department of Electrical and Computer Engineering Beer‐Sheva Israel

**Keywords:** computational imaging, deep‐learning‐DOT, diffuse tomography, time‐of‐flight

## Abstract

Imaging through highly scattering media is a challenging problem with numerous applications in biomedical and remote‐sensing fields. Existing methods that use analytical or deep learning tools are limited by simplified forward models or a requirement for prior physical knowledge, resulting in blurry images or a need for large training databases. To address these limitations, we propose a hybrid scheme called Hybrid‐DOT that combines analytically derived image estimates with a deep learning network. Our analysis demonstrates that Hybrid‐DOT outperforms a state‐of‐the‐art ToF‐DOT algorithm by improving the PSNR ratio by 4.6 dB and reducing the resolution by a factor of 2.5. Furthermore, when compared to a deep learning stand‐alone model, Hybrid‐DOT achieves a 0.8 dB increase in PSNR, 1.5 times the resolution, and a significant reduction in the required dataset size (factor of 1.6–3). The proposed model remains effective at higher depths, providing similar improvements for up to 160 mean‐free paths.
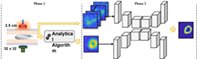

## Graphical abstract

This paper introduces a hybrid scheme called Hybrid‐DOT, which combines analytically derived image estimates with a deep learning network. The architecture of Hybrid‐DOT is X‐shaped, consisting of two parallel arms. Each arm is responsible for extracting information either from the raw data or from an analytically derived estimate. Through our analysis, we have shown that Hybrid‐DOT surpasses a state‐of‐the‐art ToF‐DOT algorithm by enhancing the PSNR ratio and improving resolution. Furthermore, the proposed model demonstrates consistent effectiveness at greater depths, delivering similar improvements for depths of up to 160 mean‐free paths.

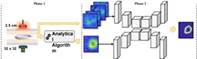

## 1 INTRODUCTION

Imaging objects using near‐infrared (NIR) lights inside a scattering medium is a tremendous challenge with countless applications ranging from biomedical imaging to robotics and autonomous driving. The main challenge hindering the possibility of exploiting non‐ionizing NIR photons is light scattering. The scattering process essentially randomizes the photon's path, which causes blurring of the imaged object and limits the system's resolution [1]. Several different approaches have been proposed to deal with this challenge, either by using ballistic gating [2, 3, 4, 5, 6], interference of light [5, 6], or by using diffusive photons [7, 8, 9, 10]. The ballistic method relies on the un‐scattered component of light which can be isolated using either time‐gating [2, 3] or coherence‐gating [4]. However, despite its many advantages its magnitude reduces exponentially with media's thickness and when the media is sufficiently thick, using it becomes impractical. Consequently, understanding how to effectively exploit the diffusive component of light, which highly dominates in many applications, became of great interest in many fields. One of the most promising techniques developed in this context is diffuse optical tomography (DOT) [1, 11, 12]. In DOT, an array of light sources illuminates the tissue, and the scattered light is measured with an array of detectors. Then, the reconstruction is done using model‐based algorithms, where one attempts to “invert” the propagation model to image the object of interest [1]. A mathematical representation of this problem can be written as follows:

(1) $$\text{Find}\;x\in X\;\text{from data}\;y=A\left(x\right)+\delta ,\;y\in Y$$

where in our case, X is the optical parameters space, Y is the measurements space, A is the propagation model of photons convolved with the optical component response and δ is the noise mechanism in the system. Generally, there are three main methods to solve this problem, they differ by their complexity, computation time and accuracy. The first two involve using a simplified forward model where the propagation model $A\left(x\right)$ is replaced with its linearized version Ax. The inverse estimate $\tilde{x}$ can be generated by transposing A or by finding an estimate to its inverse using iterative methods. The third and most effective method uses an iterative non‐linear inversion process. These methods consist of numerous updates to an initial guess by solving an adjusted linearized problem. The main strength of this method is that the linearized forward model is updated each iteration and leads to an overall better estimate of the original one. Given sufficient diversity of measurements, DOT achieves cm‐scale reconstructions (Figure 1A) even in the diffusive regime (>50 transport mean free paths—TMFP). Another important variation to traditional DOT methods is the use of temporal information or time‐of‐flight (ToF) in the reconstruction process. By using the temporal spread of the scattered photons, additional knowledge about the scattering medium is achieved, which further improves the reconstruction quality. In recent years, several groups implemented ToF‐DOT systems to image object inside or through highly diffusive medium and were able to achieve mm‐scale resolution [7, 8, 9, 10]. However, despite of the proven success of DOT and ToF‐DOT, their applicability for biomedical applications is still limited. Most DOT algorithms use relatively simple forward‐mapping to make the reconstruction process computationally feasible. This leads in many cases to an over simplified model that cannot reproduce the realistic properties of the process it aims to describe. Therefore, most existing algorithms still produce low resolution noisy images with clear artifacts, limiting their ability to detect the high‐resolution features required in biomedical imaging (Figure 1B).

**FIGURE 1:**
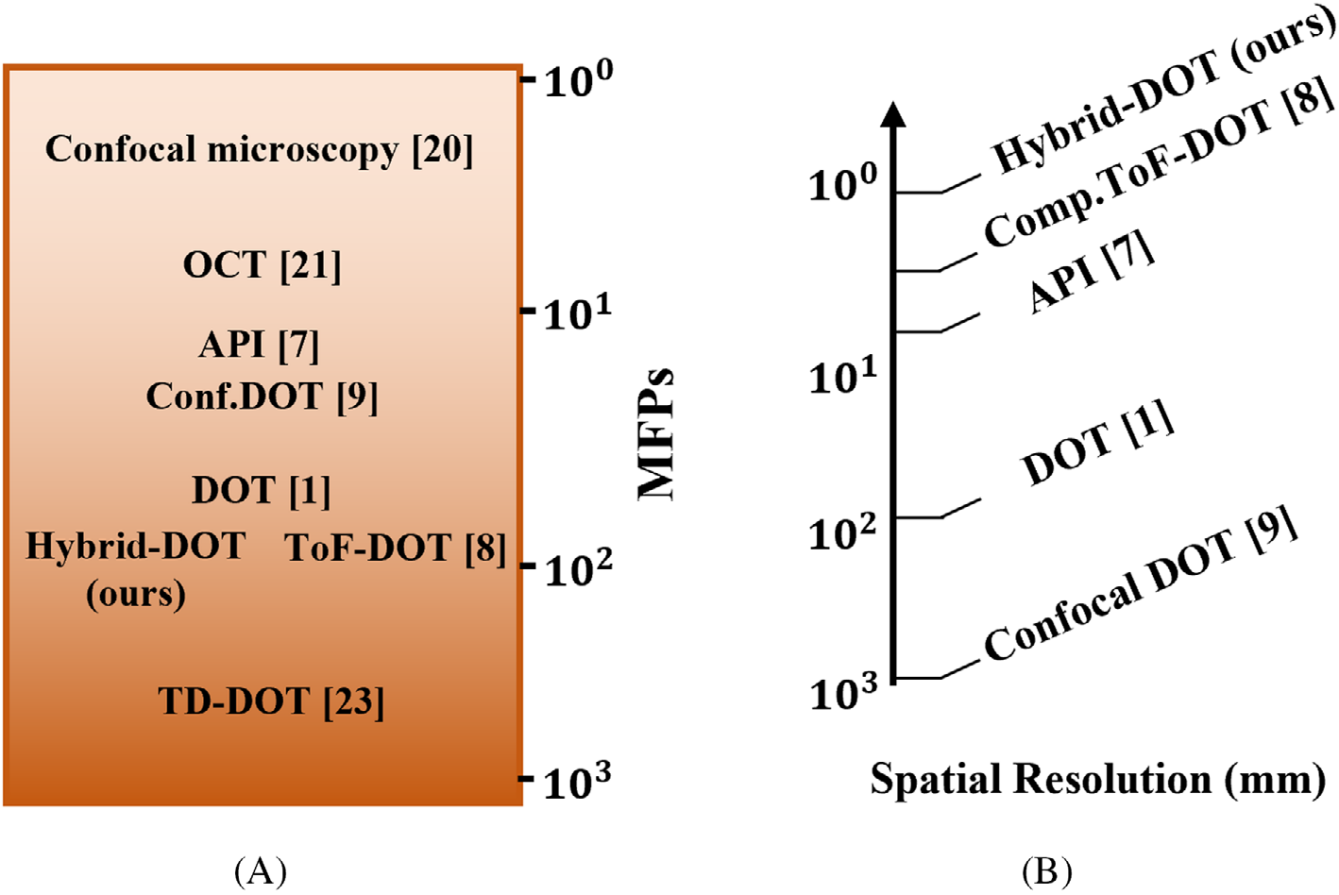
Imaging depth and spatial resolution of DOT techniques. (A) Approximate imaging depth of optical imaging techniques. (B) Approximate spatial resolution of different DOT techniques.

Another interesting approach for solving DOT reconstruction is the use of deep learning‐based algorithms. Deep learning (DL) is a purely data‐driven method used to estimate mappings between source and target distributions. DL approaches have demonstrated remarkable performance in many fields which involve inverse problem solving. These include super‐resolution [13, 14], deblurring [15, 16] and compressive imaging [17, 18]. The main strengths of deep learning are its fast inference and the fact that when given sufficient amount and diversity of data, deep learning architectures can model complex phenomena with high degree of accuracy, avoiding the simplifications made by analytical models. Despite the mentioned advantages, the power of deep learning is also its main drawback. Deep learning acts as a “black box” without prior knowledge of the underlying physics of the problem, and the inversion procedure is only indirectly learned during the minimization process. Motivated by this understanding, in this paper we propose a hybrid reconstruction algorithm combining the strengths of both approaches. The proposed algorithm is composed of two phases. In the first phase, we exclusively apply the analytical algorithm to the measurements, resulting in an initial object estimate. In the second phase, we employ a deep learning model on both the measured boundary data and the image estimate to generate a refined output. This technique allows for sequential operation on the raw data, where an initial estimate is derived and subsequently enhanced using deep learning techniques. The deep learning architecture employed is X‐shaped, featuring two parallel arms. Each arm extracts information from either the raw data or the analytically derived estimate. By combining their outputs, we obtain both the low‐resolution features from the estimate and corrections extracted from the raw data. The proposed reconstruction method and the corresponding imaging procedure are shown in Figure 2.

**FIGURE 2:**
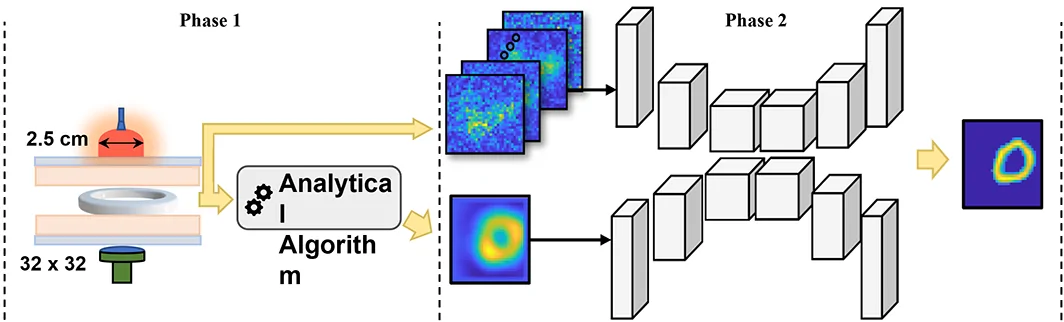
Visualization of the imaging procedure and the proposed dual‐phase algorithm (Hybrid‐DOT). In the first phase, the transmission measurements are made for a single source illuminating the object (sandwiched between two scattering slabs), the scattered light is measured by a time resolved camera (32 × 32 pixels). Then, the results are passed to an analytical algorithm to produce an initial estimate. In the second phase, both the raw data and the analytical estimate are passed to the networks to further improve the reconstruction accuracy.

Our method's advantage can be viewed from two perspectives: as a simplified reconstruction task due to the addition of the image estimate or as a learned postprocessing algorithm for the analytical reconstruction. The first perspective emphasizes that by allowing the network access to an estimated solution, the complexity of the task is reduced, leading to improved performance and a smaller required dataset size. The second perspective emphasizes that the deep learning phase serves as an adaptable postprocessing step that effectively learns to identify and remove common artifacts associated with the analytical model. Both perspectives highlight that by using a hybrid algorithm, an enhanced image can be attained. Comparison of our proposed technique results with deep learning‐based reconstruction and the analytical inversion technique indicates that (a) the achieved resolution while propagating deeply (>80 TMFPs) in the medium is improved by 60% from 10 to 4 mm, (b) the reconstructed image PSNR value improved by more than 30% from 14.41 to 19.02 dB mm, (c) the required dataset size per given accuracy level reduced dramatically by a factor of between 1.6 and 3 depending on the required accuracy (1500–500 for PSNR of 17 dB and 2500–1550 for PSNR of 18.2 dB).

## 2 MATERIALS AND METHODS

### 2.1 Incorporating the analytical model into the DL architecture

The imaging scenario we use for the demonstration of our model is described in [8]. Briefly, we aim to reconstruct a 2D object that is sandwiched between two 2.5 cm slabs of homogenous scattering material (Figure 2‐left). A laser with a 2.5 cm radius spot size is used to illuminate the tissue, and some of the transmitted light is absorbed by the hidden object. The transmitted light is then collected by a 32 × 32 array of time‐resolved pixels with a 55 ps timing resolution, which allows for the collection of 3D data with a spatial resolution of 32 × 32 pixels and a temporal resolution of 230 × 55 ps time bins. These measurements will be used as the raw data for reconstruction. The parameters used for both forward and backward calculations are provided in Table 1.

**TABLE 1:** Simulation parameters used for both the forward calculation and the inversion algorithm.

Illumination width (cm)	Scattering TMFP (cm)	Absorption MFP (cm)	Sample width/MFPs	Object dimension/pixel number	Temporal range/resolution	Iteration number
2.5	0.06	11.1	5–10 cm/80–160	3.2 × 3.2 cm^2^/32 × 32	0–5 ns/50 ps	0–2K

As explained above, deep learning models can be seen as “black boxes” because they do not have prior knowledge of the underlying physics of a problem or of the imaging setup. This means that a large amount of high‐quality data is needed to model and invert complex phenomena, which is often scarce in biomedical applications. To address this issue, some researchers have attempted to incorporate physical models into deep learning architectures. One approach involves reformulating the problem as denoising or deblurring an estimated inverse of the original image. This can be represented mathematically as follows:

(2) $$\begin{array}{c} F_{\theta }=G_{\theta }\left(\tilde{x}\right)+K_{\theta }\left(y\right) \end{array}$$

where $G_{\theta },K_{\theta }$ are the optimized network trained on the estimated inverse $\tilde{x}$ derived by using physical model and the measurement vector y, respectively. The two arms $G_{\theta },K_{\theta }$ are used to improve the initial estimate by adding high resolution features and remove noise and artifacts from the original image. This approach was studied and applied in various imaging applications, such as compressive sensing and CT scanning [17, 19, 20], however its use for DOT it still in its infancy.

### 2.2 Network architecture

In this work, we propose a novel method called the Hybrid‐DOT network, which is presented in Figure 3 and is based on the U‐Net structure proposed by Ronneberger et al. [21]. The architecture is X‐shaped, featuring two parallel arms. Where each arm extracts information from either the raw data or an analytically derived estimate. The dual U‐Net approach is beneficial since it can extract the necessary information from each input without restricting improvement to the original image estimate alone. Each basic U‐Net building block is composed of n down‐blocks followed by n up‐blocks. In each down‐block, a series of convolution, dropout and ReLU layers are applied followed by a max pooling operation. Resulting in increased channel number and reduced spatial dimension. The up‐blocks consists of up‐conv, concatenation, and convolution layers to extract the information from both the previous up‐block and the down‐block with the same n. The output of the U‐Net is achieved by applying a final 1D conv layer to achieve a 2D image. An important modification is made on the raw data U‐Net arm. U‐Nets perform optimally when they have symmetric input–output dimensions, however in our case the input is 3D in nature, with two spatial and one temporal dimensions, and a 2D output, representing the imaged object. To mitigate this problem, we introduce an initial 3D to 2D layer to the network to derive an optimal weighted sum across the ToF frames and obtain an informative 2D input. Moreover, the initial mapping reduces the computational overload of our algorithm and dramatically decreases its runtime. There are two distinct training phases for our algorithm, in the first (DL‐DOT) a stand‐alone DL network is applied only on the raw measurements to generate the reconstruction. In the second stage (Hybrid‐DOT), analytical estimates are introduced and leveraged for a better reconstruction. In order to utilize the DL‐DOT learning stage for the Hybrid‐DOT training, an early time‐gated image is used for the DL‐DOT stage instead of the analytical estimate which is only introduced later during the hybrid training.

**FIGURE 3:**
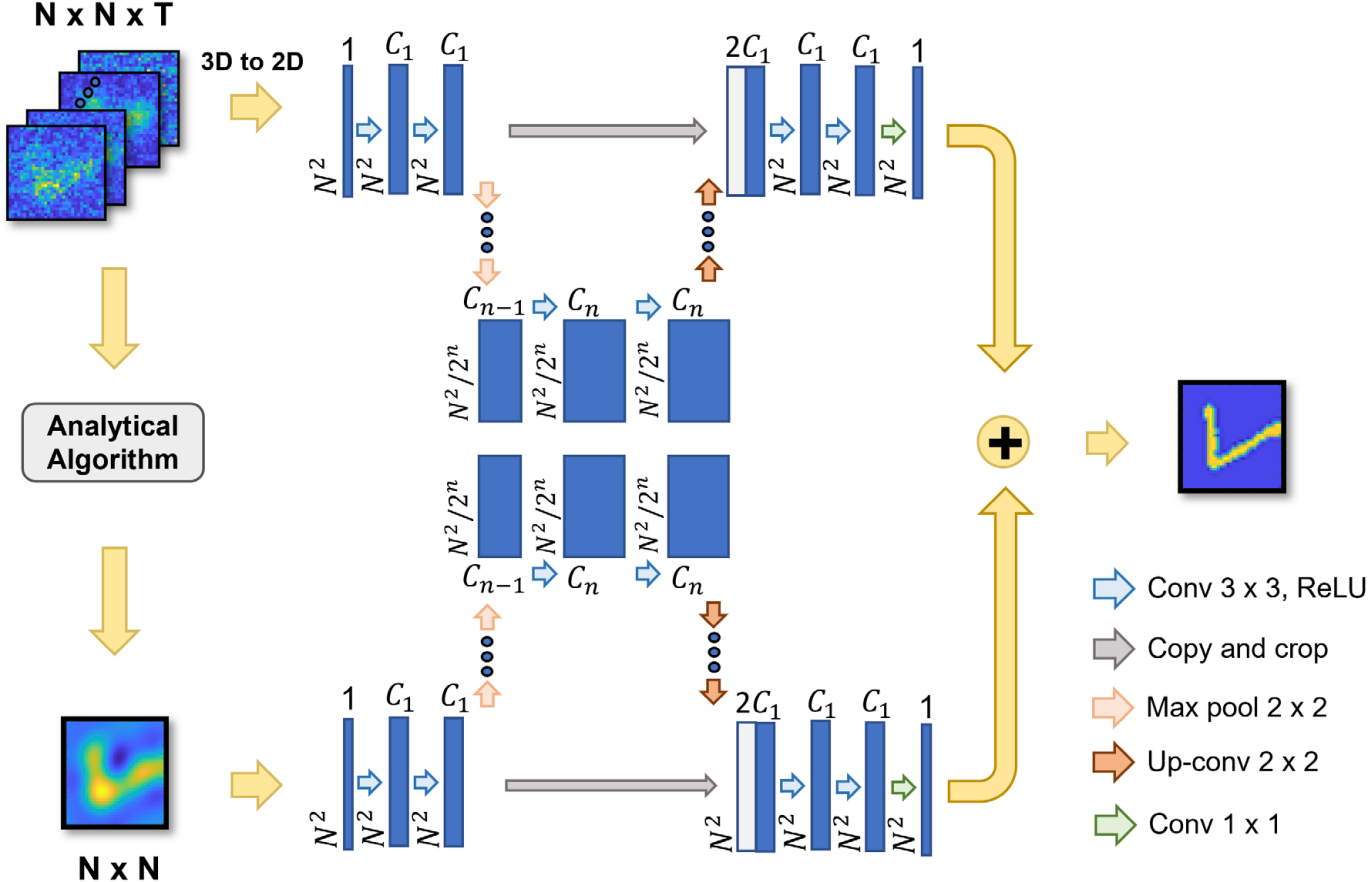
Illustration of the U‐Net network. Two U‐Nets are operated in parallel. The outputs from both networks are summed together at the output. $C_{n}$, *N*, *T* are the channel number, spatial pixel number and time bins, respectively.

### 2.3 Dataset generation

To ensure a realistic comparison between our proposed method and existing analytical time‐of‐flight diffuse optical tomography (ToF‐DOT) algorithms, we evaluated their performance under the same conditions. To this end, we modified the algorithm developed by Lyons et al. [8] to generate the dataset used to train our network and as the benchmark for evaluating our proposed algorithm. The code and corresponding geometry used for this process are described in detail in [8]. The generation pipeline is illustrated in Figure 4. First, we used the MNIST dataset [22] as the mask data that the network aims to reconstruct. To diversify the data, we applied augmentation to vary the mask sizes and orientation. Second, we applied the modified code on each mask to obtain the time‐resolved scattering measurements associated with each mask along with an early time‐gated version of the data, these will serve as the input of the network. Third, for each mask‐boundary measurement pair, we obtained a corresponding analytical reconstruction using the inversion algorithm developed in [8]. We saved the results for 0–2K iteration steps to use as the benchmark and as the analytical estimate input for the Hybrid‐DOT network. We also applied the same procedure for mediums with varying depths to understand the limitations of our algorithm. The parameters used to simulate the boundary measurements and generate the image estimate are presented in Table 1.

**FIGURE 4:**
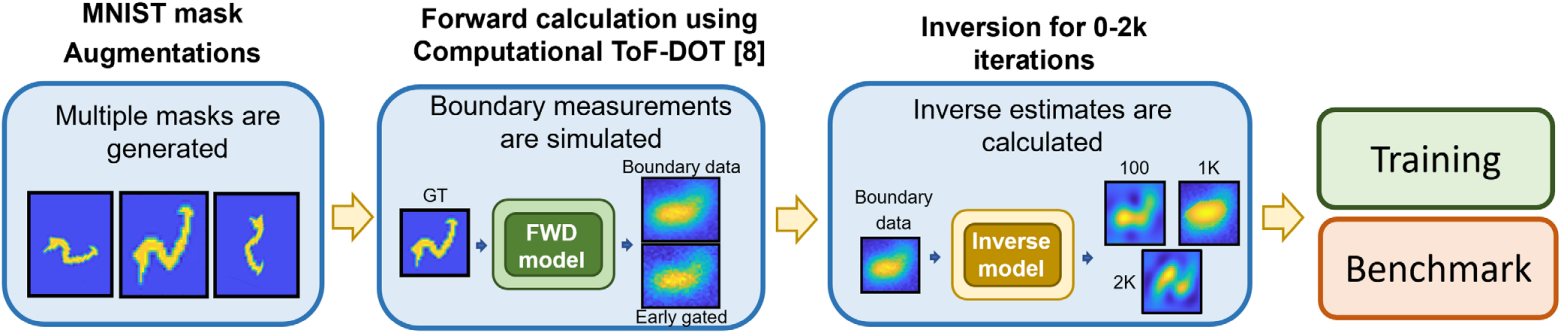
Dataset generation pipeline. First, augmentation are made on MNIST masks. Then for each mask the scattering measurements are calculated using the Comp. ToF‐DOT forward model. Finally, the boundary measurements are used to reconstruct the original image that will be used as the network input and as the benchmark of the proposed algorithm.

### 2.4 Training procedure

The model training process was divided into two phases. In the first phase, we trained the DL‐DOT network without the analytical estimate using boundary and early‐gate data. To optimize the training conditions, we evaluated various architectures, hyperparameters, and preprocessing schemes. Our primary goal in this phase was to benchmark the network performance on with the stand‐alone DL model, and pre‐train the model weights for the Hybrid‐DOT algorithm. In the second phase, we trained the Hybrid‐DOT algorithm with the same training conditions as the DL‐DOT network, but with the analytical estimate added. To leverage information from the first phase and further enhance the performance of the Hybrid‐DOT algorithm, we employed transfer learning by initializing the model weights using the results of the previous phase. To prevent over‐fitting, we performed data augmentation techniques such as random horizontal flip, random rotation, and random scaling on the training dataset. We used the Adam optimizer and evaluated the models using the peak signal‐to‐noise ratio (PSNR) metric. We trained the models using Huber loss and randomly split the dataset into training, validation, and test data with 80%, 10%, and 10% respectively. All trainings were conducted on a GPU workstation with two Nvidia GeForce RTX 2080 Ti GPU with 11 GB RAM.

## 3 RESULTS

### 3.1 DL‐DOT architecture

In this section, we aim to determine the optimal hyperparameters for the stand‐alone DL network (DL‐DOT). The DL‐DOT training phase will help us to understand how to design the hybrid network and will also serve as a baseline reference for the hybrid algorithm. The DL‐DOT network is composed of two parallel U‐Net, each operating on either the raw data or on an early‐gated image. To achieve optimal performance, several comparisons were made during the architecture design phase: (a) varying the number of U‐Net layers, (b) varying the number of the temporal bins used in the spatio‐temporal input to the network, (c) using or discarding the raw data “arm”. We have also verified the benefits of using the dual U‐Net approach (Table S1, Figure S1) and the usage of an initial 3D–2D mapping of the input (Table S2). A detailed performance assessment of the different setups is presented in Table 2.

**TABLE 2:** PSNR values for different setups, the best value from each step was used as the base parameter for the next variation.

Method	PSNR values for different setups (dB)			
Block number—1/2/3	16.25	18.27	**18.63**	−
Bin number—10/20/30/40	17.11	18.32	**18.63**	18.15
Raw data usage—w/w.o.	**18.63**	17.95	−	−

Based on the results presented in Table 2, the optimal training conditions for the DL‐DOT algorithm were identified as using 30 time‐bins, 3 blocks, and incorporating the boundary data. To evaluate the utility of this approach, three different metrics were used for comparison against the benchmark method: average PSNR on the test dataset, minimum length resolvable by the algorithm, and computation time. To assess our method's capability to differentiate closely located points, we followed the methodology described in prior studies [7, 23]. These papers utilized a wedge‐shaped inclusion and examined the reconstructed image to establish the system resolution. Therefore, we adopted this approach, enabling us to conduct a consistent comparison of the different methods. The results of the different metrics are summarized in Table 3, with additional information presented in Figure 5, which includes graphs of accuracy, resolution, and reconstruction speed for both the baseline DL method and the analytical algorithm. The resolution images used to define the resolvable length of each method are also included in the figure.

**TABLE 3:** Quantitative comparison of [8] and our method in terms of PSNR, resolution and runtime for varying iteration numbers.

Measure	Lyons et al. [8], # of iterations			DL‐DOT (ours)
	500	1K	2K	
PRNR (dB)	14.04	14.31	14.41	**18.22**
Resolution (mm)	12	11	10	**6**
Runtime (s)	60	120	240	**9E−3**

**FIGURE 5:**
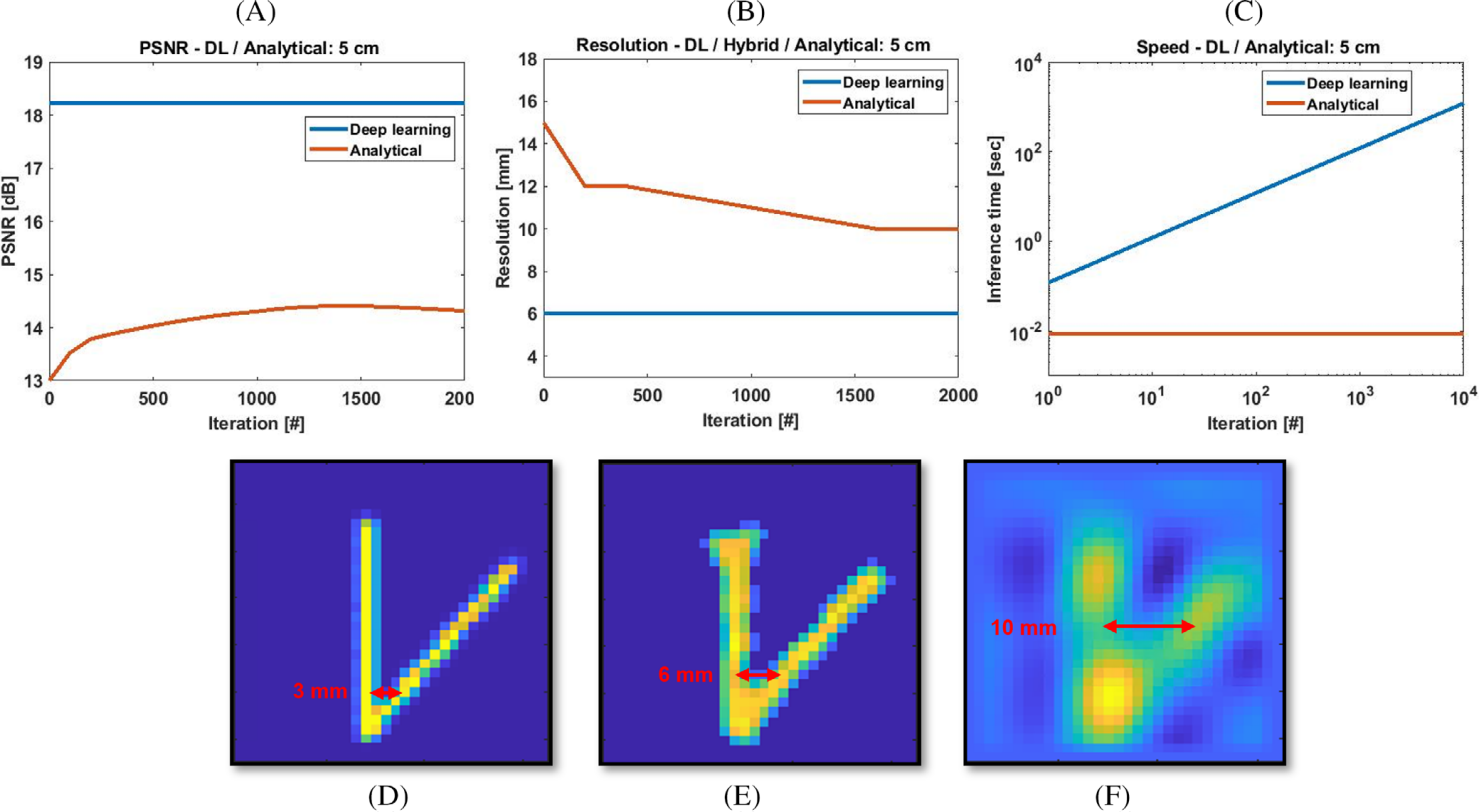
Comparison of DL‐DOT to [8] for varying iteration number. (A) PSNR values for iterations 0–2K. (B) resolution values for iterations 0–2K. (C) runtime values for iterations 0–2K. (D–F) resolution images used to define the minimum recoverable length (red)—GT, DL‐DOT, analytical, respectively.

### 3.2 Hybrid‐DOT architecture

In this section, the analytical prior estimate arm is added. Hence, the network trains to reconstruct the image using both the boundary measurement and the analytical estimate. The additional information fed into the network simplifies the complexity of the task and is expected to improve the reconstruction accuracy and to reduce the dataset size required for training. We would like to assess the Hybrid‐DOT performance compared with both ToF‐DOT algorithms and with the stand‐alone DL‐DOT model. Three different metrics were chosen. The mean PSNR of the three approaches for varying dataset sizes (500–2500 samples), their resolution and their runtime. In Table 4, quantitative comparison of the Hybrid‐DOT, DL‐DOT and ToF‐DOT for varying iteration numbers and dataset sizes are shown.

**TABLE 4:** Quantitative comparison of DL‐DOT, Hybrid‐DOT and ToF‐DOT methods in terms of PSNR, resolution and runtime for varying iteration numbers and dataset sizes.

Measure	Lyons et al. [8], # of iterations	DL‐DOT	Hybrid‐DOT, # of iterations		
	2K		500	1K	2K
PSNR at 500 samples	14.41	15.83	16.55	16.71	**17.01**
PSNR at 1.5K samples	14.41	17.24	17.38	17.85	**17.99**
PSNR at 2.5K samples	14.41	18.22	18.59	18.96	**19.02**
Resolution (mm)	10	6	4	4	**4**
Runtime (s)	240	9E‐3	60	120	240

In Figure 6 graphs for the different methods in terms of accuracy and resolution are shown, additionally we compare the PSNR versus iteration number of the baseline DL method and the hybrid algorithm used with different iterations.

**FIGURE 6:**
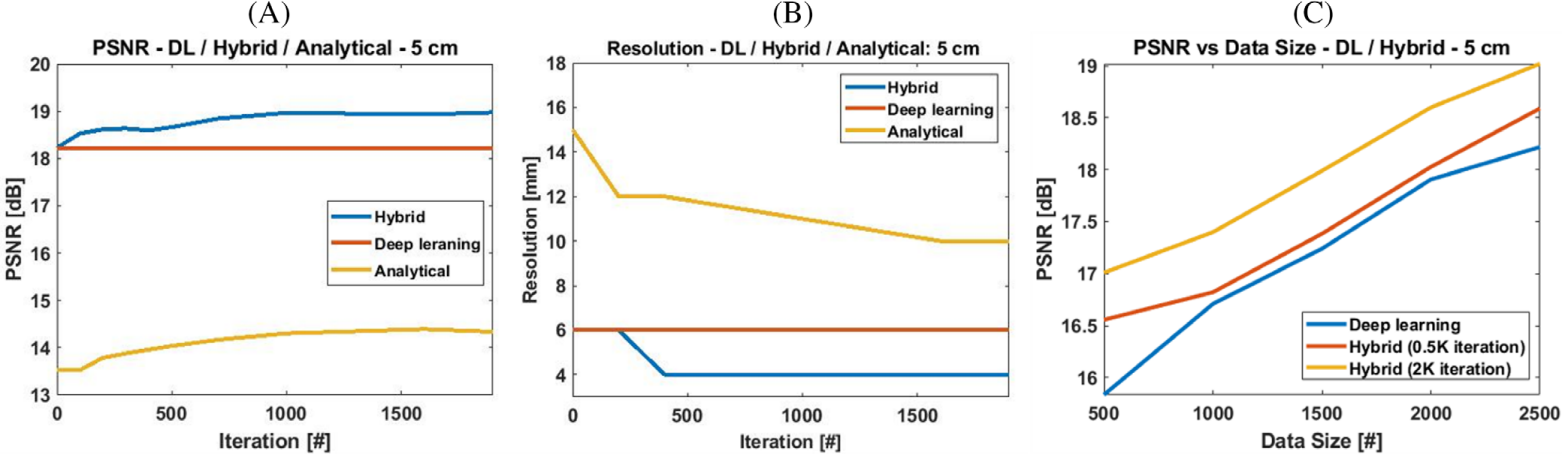
Comparison of Hybrid‐DOT, DL‐DOT and [8] for varying iteration number and dataset sizes. (A) PSNR values, (B) resolution values, (C) PSNR values of Hybrid‐DOT and DL‐DOT for varying dataset sizes, 0.5K and 2K stands for the number of analytical iteration steps used to generate the image estimates.

### 3.3 Extension to higher depths

In previous sections, we demonstrated the capability of both DL‐DOT and Hybrid‐DOT methods to image objects within highly scattering media with a 5 cm width, equivalent to over 80 TMFP. However, in this section, we investigate the limitations of these techniques in terms of imaging depth. To achieve this, we utilized the dataset generation protocol described in the previous sections to simulate forward boundary measurements and analytical reconstructions for objects embedded in media with depths of 7.5 and 10 cm, equivalent to 120 and 160 TMFP, respectively. We then used these datasets to train both our networks and the analytical algorithm. The quantitative results obtained for these deeper mediums are summarized in Table 5, and exemplary images are provided in Figure 7.

**TABLE 5:** Quantitative comparison of DL‐DOT and Hybrid‐DOT methods in terms of PSNR and resolution for varying iteration numbers and varying depths.

Measure	Depth	Lyons et al. [8]			DL‐DOT	Hybrid‐DOT		
		500	1K	2K		500	1K	2K
PRNR (dB)	5 cm	14.04	14.31	14.41	18.22	18.59	18.96	**18.99**
	7.5 cm	13.09	13.18	13.62	16.43	17.53	17.62	**17.78**
	10 cm	12.12	12.25	12.57	15.95	16.83	16.90	**17.24**
Resolution (mm)	5 cm	12	11	10	6	4	4	**4**
	7.5 cm	15	13	12	8	7	5	**5**
	10 cm	15	15	15	9	7	6	**6**

**FIGURE 7:**
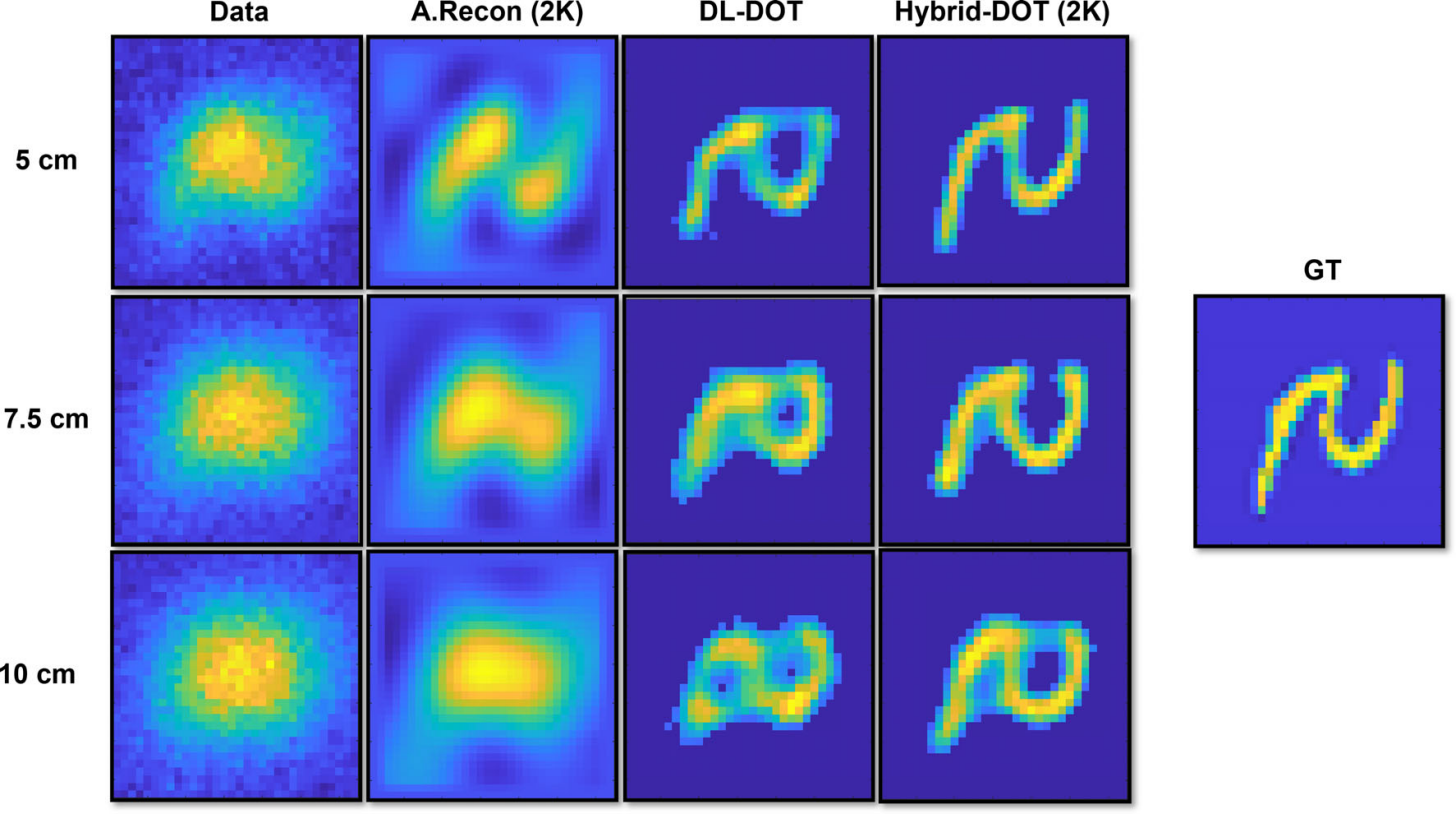
Exemplary reconstruction results of the analytical algorithm (2K iteration), DL‐DOT and Hybrid‐DOT for varying medium depths (5–10 cm).

## 4 DISCUSSION AND CONCLUSION

In this paper, we assessed the possibility of combining analytical methods together with deep learning‐based reconstruction to image objects embedded inside turbid media. The goal was to develop an algorithm which can produce clean and accurate images with a reduced number of training samples. By analyzing the results presented above several conclusions can be made regarding the optimal architecture design. First, it was discovered that incorporating raw data along with the initial estimate resulted in improved performance. This indicates that the additional information is effectively utilized in the network inference pipeline. Second, using a compressed version of the TOF information instead of the full temporal data proved to be beneficial. This was achieved by binning the temporal data and employing an initial 3D–2D mapping of the spatio‐temporal input. The improvement can be attributed to the simplification of the input data through the initial mapping. This methodology build upon previous research [24, 25], which leveraged selective moments of the temporal information for reconstruction instead of the complete time resolved data. Furthermore, the ability of the network to learn an optimal weighted sum across the TOF data reduced computational burden and memory requirements while simultaneously enhancing reconstruction accuracy compared to using the full temporal information. Finally, in the design stage of the DL‐DOT system, it was found that a dual U‐Net design was optimal. This success can be attributed to the parallel networks’ ability to extract necessary information from each arm without restricting the network's improvement solely to the image estimate as it is.

When comparing the Hybrid‐DOT model to the analytical algorithm, significant increase in terms of both the PSNR and resolution were registered. The PSNR was improved by 4.8 dB (<133%) and the resolution was improved by 6 mm (60%). Similar improvements were registered for higher depths. A 4.7 dB/7 mm improvement was achieved for 7.5 cm medium depth and 5.1 dB/9 mm improvement was achieved for 10 cm medium depth. The second comparison was made with the stand‐alone DL‐DOT model, which does not use the analytical estimate in the reconstruction process. The Hybrid‐DOT model achieved a 0.8 dB PSNR and 2 mm resolution improvement over the DL‐DOT method for 5 cm medium. Additionally, 1.35 dB/3 mm and 1.3 dB/3 mm improvements were registered for 7.5 and 10 cm, respectively. These results prove that the Hybrid‐DOT approach is superior in terms of accuracy to both the DL and the analytical approach, even for higher medium dimensions. The second consideration was evaluating if the use of the analytical estimate for the Hybrid‐DOT reconstructions reduces the required dataset size for a given accuracy. To show this qualitatively, a plot of the model accuracy versus the dataset size was given for the DL‐DOT and the Hybrid‐DOT (with 500 and 2K analytical iteration steps). When the Hybrid‐DOT (2K analytical steps) is compared to the DL‐DOT model, the required dataset size per accuracy was reduced by a factor of between 1.6 and 3 (1500–500 for PSNR of 17 dB and 2500–1550 for PSNR of 18.2 dB). This is essential since the main bottleneck of current dl‐based tools is the high cost of generating large and consistent training databases. It was also demonstrated that for all dataset sizes the model accuracy increased when the number of analytical estimate steps increased as well ($\mathrm{PSN}R_{0-\text{iterations}}<\mathrm{SN}R_{500-\text{iterations}}<\mathrm{PSN}R_{2K-\text{iterations}}$). However, this was at the cost of an increased runtime. Therefore, the idea of using a hybrid scheme combining analytical and DL approaches introduces a trade‐off in terms of accuracy and runtime, where different applications can choose their optimal operation conditions. We expect that or biomedical applications that prioritize reconstruction accuracy and have a limited number of experimental samples, this addition can be considered an advantage. However, in real‐time applications such as robotics and autonomous driving, it may be viewed as a disadvantage. Moreover, our analysis proves that the highest gain is apparent for lower number of iterations. This is comforting since the gain from the increasing the iteration number plateaus quite quickly and not that many steps are required to significantly improve the accuracy of the results and gain from the added information. In conclusion, the combination of these two commonly used methods is still in its juvenile form. Our analysis suggests that the hybrid approach has the potential to be a useful tool in biomedical applications, where reconstruction accuracy is crucial and dataset size is limited. However, further research is needed to fully understand and verify the effectiveness of this approach experimentally.

## FUNDING INFORMATION

Israeli Ministry of Science, Technology and Space; Ben Gurion University of the Negev internal student fellowship.

## CONFLICT OF INTEREST STATEMENT

The authors declare no conflicts of interest.

## Supporting information


**Appendix S1:** Supporting Information.

## Data Availability

The availability of the training data is of great importance for deep learning based biomedical research. An extensive simulated dataset consiting of the forward measurements as well the analytical reconstruction was generated for more than 2000 different samples. Therefore, the datasets involved in this article can be shared upon reasonable request.
