# Supplementary material for: Imaging inside highly scattering media using hybrid deep learning and analytical algorithm
Source: J Biophotonics. 2023 Jul 19;16(10):e202300127. doi: 10.1002/jbio.202300127 (PMC13631377; doi:10.1002/jbio.202300127)
Supplement: Supplementary file 1 [file JBIO-16-e202300127-s001.docx]

**Supplementary information**

**Imaging inside highly scattering media using hybrid deep learning and analytical algorithm**

Ben Wiesel, Shlomi Arnon

Ben-Gurion University of the Negev, P.O Box 653 IL-84105 Beer-Sheva, South district, ISRAEL.

# 1. DL-DOT architecture design analysis

This section aims to provide supplementary information about our analysis aimed at determining the optimal deep learning architecture and hyperparameters. We present two key comparisons to understand the effectiveness of the initial 3D-2D mapping and the usage of the dual U-Net architecture.

## 1.1. Parallel network design

During the architecture development phase, we explored different U-net configurations: a) a single U-Net applied only on the raw data (Fig S1, left), b) single U-Net applied on a concatenated version of the raw data and image estimate (Fig S1, middle), c) using two parallel arms each operating on either the raw data or image estimate (Fig S1, right). A detailed performance assessment of the different setups is presented in Table S1.

**Table S1: PSNR values for the single/dual U-Net design**

| Method | PSNR |
| --- | --- |
| Single – raw data | 15.92 |
| Single – Concatenated | 16.43 |
| **Double U-Net** | **18.63** |

The dual U-net approach demonstrated a significant improvement in reconstruction accuracy, making it the selected network for our study.

Figure S1: The three setups used to evaluate the single/double U-Net design

## 1.2. Learnable 2D-3D mapping

During the development phase of the architecture, we explored various approaches to handle the temporal information derived from the measurements. To reduce the computational and memory requirements of our model, we adopted the approach suggested by previous studies [1, 2]. Instead of using the complete time-resolved data, we leveraged selective moments of the temporal information for reconstruction. To implement this, we introduced an initial 3D to 2D layer in the network, which allowed us to derive an optimal weighted sum across the time-of-flight (ToF) frames, resulting in an informative 2D input. To validate our choice and ascertain whether meaningful information was lost during the compression stage, we compared the performance of our proposed algorithm with a 3D U-Net architecture, which can directly handle the input data without the initial mapping. A comprehensive evaluation of the performance of the different setups is presented in Table S2.

**Table S2: PSNR values for the 2D/3D U-Net design**

| Method | PSNR |
| --- | --- |
| **2D U-Net** | **18.63** |
| 3D U-Net | 16.85 |

The initial 3D-2D mapping along with the 2D U-Net approach demonstrated a significant improvement in reconstruction accuracy, making it the selected network for our study.

# 2. Supplementary bibliography

[1] L. Hervé, A. Puszka, A. Planat-Chrétien, and J.-M. Dinten, “Time-domain diffuse optical tomography processing by using the Mellin–Laplace transform,” *Appl Opt*, vol. 51, no. 25, p. 5978, Sep. 2012.

[2] H. Wabnitz, D. Contini, L. Spinelli, A. Torricelli, and A. Liebert, “Depth-selective data analysis for time-domain fNIRS: moments vs. time windows,” *Biomed Opt Express*, vol. 11, no. 8, p. 4224, Aug. 2020.
